# Barriers and facilitators of nurse-led self-management support for adolescents with epilepsy: A mixed-methods study in transition preparation

**DOI:** 10.1016/j.heliyon.2024.e33774

**Published:** 2024-06-26

**Authors:** Cui Cui, Shuangzi Li, Hengyu Zhou, Wenjin Chen, Changmin Xiao, Mingping Fan, Xianlan Zheng

**Affiliations:** a Department of Nursing, Children's Hospital of Chongqing Medical University, National Clinical Research Center for Child Health and Disorders, Ministry of Education Key Laboratory of Child Development and Disorders, Chongqing Key Laboratory of Pediatrics, Chongqing, 400014, China; b Department of Neurology, Children's Hospital of Chongqing Medical University, National Clinical Research Center for Child Health and Disorders, Ministry of Education Key Laboratory of Child, Chongqing, 400014, China; c Development and Disorders, Chongqing Key Laboratory of Pediatrics, Chongqing, 400014, China; d School of Nursing, Chongqing Medical University, Chongqing, 400014, China

**Keywords:** Adolescent, Epilepsy, Evidence-based practice, Mixed-method research, Pediatric nursing, Self-management

## Abstract

**Aims:**

To gain insight into the readiness for evidence-based practice of self-management support during transition for adolescents with epilepsy among pediatric nurses, and to explore the promoting and hindering factors.

**Design:**

A mixed-methods design with an explanatory sequential approach was employed.

**Setting:**

Three specialty children's hospitals in southwest regions of China.

**Methods:**

In phase 1, a total of 126 participants were included in the Survey of Clinical Readiness of Evidence-Based Nursing Assessment (CREBNA) from Dec 2022 to Feb 2023. Total scale and subscale scores were calculated. In phase 2, we developed the interview outline based on the analysis of the quantitative results. In-depth interviews (n = 15) were conducted from Feb 2023 to Apr 2023 to explain and supplement the quantitative phase results.

**Results:**

The total score of CREBNA indicated that teams have good readiness and capacity for implementation of evidence-based nursing practice Compared with the norm. The scores of the three subscales of evidence factors, organizational environment, and promoting factors were normal. In subsequent interviews and integration, we extracted four themes based on the Knowledge-To-Action (KTA) framework: 1. organization barriers (incentive mechanism, interdisciplinary cooperation process, information aids); 2. operational barriers (Exemplary evidence-based practice, time pressure, gaps in patient and family understanding of evidence); 3. individual-level barriers (evidence-based and professional knowledge reserve, professional autonomy, shared decision-making roles, dependence on habitual clinical behaviors); and 4. facilitating factors (leadership commitment, self-management identity, transition service needs, patient- and family-centered care culture). A conceptual model was constructed based on the KTA.

**Conclusion:**

It is feasible to carry out evidence-based practice of nurse-led self-management support in transition preparation. Nursing researchers and managers should carry out knowledge selection and tailoring based on barriers at the organizational, operational, and individual levels to promote favorable factors and improve the smooth transition of adolescents with chronic diseases.

## 1 Introduction

Epilepsy is one of the most common neurological diseases. There are nearly 10 million epilepsy patients in China, two-thirds of whom are adolescents under 18 [1,2], and about 50 % of seizures will be carried through to adulthood [3]. Adolescents with epilepsy (AWEs) have a complex, stage-specific disease and are often family-dependent. Inadequate or poor preparation for the medical transition from childhood to adulthood can lead to poor seizure control, social development difficulties, and sudden death [4,5]. Medical transition is the planned transition of adolescents with chronic diseases from a child- and family-centered pediatric environment to an adult medical environment [6]. transition preparation, as an essential element of the medical transition process, refers to the ability of adolescents with chronic diseases, and their support systems (family, medical providers, and society), to begin, continue, and complete medical care transfer [7]. Self-management is a crucial development skill in transition preparation for adolescents with chronic diseases [8]. Drawing upon a body of research on self-management, we define this as the ability of children and young people and their families to manage to live with epilepsy, using the core self-management skills of problem solving, decision making, resource use, and taking action to establish a dynamic and continuous process of self-regulation [9]. The aim is to improve seizure control, well-being, and quality of life.

It is a formidable task for pediatric nurses to practice self-management projects during the transition process for adolescents with neurological diseases [9,10]. First, self-management practices aligning with adolescents’ cognitive, physical, and mental requirements are essential to delivering patient-centered care [11]. Second, the level of health care, medical resources, and transition service status of a location also need to be considered. Third, unanticipated episodes of illness may cause regression in self-management skills, which requires repeated intervention [12]. The impact of epilepsy includes the unpredictability of seizures, the complicates treatment regimens (e.g., diet, medication), the psychosocial comorbidities, and the physical development. It also creates stigma issues, which are more evident during adolescence [13]. Therefore, it is very challenging for AWEs to develop their self-management skills during transition preparation [10]. AWEs and their families need professional support to engage in self-management activities to meet the challenges of the disease.

The healthcare provider should offer self-management support to patients with epilepsy [14]. The key in home- and community-based treatment for adolescents with chronic diseases is their overall self-management, which strengthens the depth, diversity, and scope of healthcare transition and contributes to the early development of self-management ability [8,15]. In transition preparation, nurses play a central role in supporting patients and families. Nurses are vital in identifying behavioral changes of adolescents with chronic diseases and their families, continuously optimizing their coping skills, and maintaining good relationships with patients. Their unique professional knowledge plays an essential complementary role in the participation of patients and families in health-related decision-making [8]. Under the multidisciplinary team care model, the role of pediatric nurses in self-management support has become increasingly prominent. Studies have shown that nursing staff, through their leadership and coordination of self-management during transition, can help patients clarify problems and guide final decisions, which can save the cost of visits and is appreciated by patients and teams [8,12].

In addition, as transition service platforms in China are not yet developed, there is little systematic support for studying transition services across the Health Care Transition spectrum. A self-management intervention involves interaction between AWEs and their families that can meet the transitional service needs of adolescents and families with chronic diseases in the situation of limited medical resources, and it can potentially reduce service costs. So far, few studies have focused on the benefits of behavioral health interventions to the healthcare system and community. Development of high-quality pediatric epilepsy self-management interventions depends on best evidence-based practice [9].

Therefore, there is an urgent need to develop clinical practice based on relevant evidence of self-management support. To improve the self-management ability of adolescents with epilepsy in transition preparation, our research team carried out the current situation study of children and families of the main interest population and the preliminary studies of an evidence-based nursing practice plan [16,17]，including an evidence summary for self-management in transition preparation from adolescents to adults with epilepsy [18], which was based on the SMART theory (Social-ecological model of adolescents and young adults' readiness for transition). Therefore, this study adopted convergent mixed methods to explore the facilitators and obstacles of preliminary evidence-based practice. Quantitative research allows for the collection of large amounts of data on the clinical readiness of evidence-based nursing. On the other hand, semi-structured learning enables a deep understanding of individuals' experiences, perspectives, feelings, and the specific contexts and experiences related to self-management support. Integrating these two methods helps comprehensively understand self-management support's impact on adolescents with epilepsy and provides more comprehensive recommendations for improving practice.

## 2 Materials and methods

### 2.1 Study design

A mixed-methods sequential explanatory design with an inductive approach was employed [19]. Qualitative data was collected to explain the quantitative results in more depth. Phase 1 aims to utilize quantitative data to validate and clarify the barriers and facilitators of nurse-led self-management support, as identified in the qualitative study. The methodology involves conducting a cross-sectional survey, wherein the CREBNA questionnaire is distributed to pediatric nurses specializing in neurology across three specialized children's hospitals between December 2022 and February 2023. This survey's primary goal is to identify obstacles to evidence-based practice effectively. Following data collection, full-scale and sub-scale scores are calculated, and item scores within each dimension are ranked. Sub-scale scores are computed individually for three dimensions: the Evidence subscale, the Organizational environment subscale, and the Facilitating factors subscale. The full-scale score is then obtained by summing up the scores from these three sub-scales. In phase 2, the participants involved in the interviews were recruited from February 2023 to April 2023. This phase was a qualitative exploration using semi-structured interviews and a combination of inductive and deductive methods [[19], [20], [21]] to generate contextualized understanding of self-management support. We integrated at both the design and methods level through connecting [21], whereby the results from the quantitative phase informed the types of providers recruited for the qualitative phase. Furthermore, we implemented integration throughout the building; we used the quantitative data to refine our interview questions and develop our deductive codes [19,21]，showing an overview of the study methodology [21] (Supplementary Figure 1).

### 2.2 Setting

The hospital conducting the research led this multicenter research project. The local Commission funded the project. In addition, two specialized children's hospitals in Sichuan and Guizhou in China were selected, both of which are guidance hospitals of the China Antiepileptic Association and the bases for the promotion and application of this project.

### 2.3 Participants

In the quantitative study phase, we used a convenience sampling method. The inclusion criteria for the pediatric nurses were: participating in caring for children and adolescents with epilepsy and voluntary participation in this study. Exclusion criteria: nurses not on duty due to vacation; training rotation nurses and student nurses. In the qualitative study phase, we used purposive sampling to select participants who were willing and able to express their own experiences, considering the maximum variability in terms of factors such as education, age, work experience, clinical position, and evidence-based practice experience based on the results of the quantitative study phase [22].

## 3 Data collection

### 3.1 Quantitative phase

General information questionnaire. The researchers designed the questionnaire to obtain general information including gender, working years, position, education background, scientific research experience, understanding and cognition of carrying out evidence-based practice (Supplementary Table 1).

Clinical readiness of evidence-based nursing assessment (CREBNA) [23]. The tool is based on the PAPIHS model, formed through a rigorous scale development procedure to help effectively identify barriers to evidence-based practice [24]. Three subscales and 31 items were included. The evidence subscale had 12 items to evaluate the applicability of evidence introduced into clinical practice. The organizational environment subscale had 9 items to evaluate leadership style and organizational culture. The 10-item facilitator's subscale assessed individual, team, and managerial facilitators to practice. A Likert 5-point scoring method was adopted, with 1 point representing completely inconsistent and 5 points representing entirely consistent. The higher the score, the better the preparation in the current clinical situation, and the more conducive to the development of evidence-based projects. The Cronbach's α coefficient of the total scale was 0.959, and for three subscales were 0.940, 0.933, and 0.915 [24] (Supplementary Table 2).

Questionnaires were collected in pediatric neurological wards in three medical institutions. The researchers trained the investigators in charge of this study. The investigators were nursing managers of the hospital with more than 12 years of service and good communication skills. The researchers provided unified instructions for filling out the questionnaire and links to access it to the participants. The investigators explained the purpose, significance, survey methods, harmlessness, and anonymity of the study to the participants and obtained consent. The items pediatric nurses did not understand were explained by investigators, and the explanation of each item was consistent. The sample size allowed for 10 % dropped samples. The calculation formula is as follows: n = (Z*α*/2*σ*/*δ*)^2^, considering *α* = 0.05 [25]; Z*α*/2 = 1.96 based on a previous study [26]. At least 122 samples are required.

### 3.2 Qualitative phase

A face-to-face, semi-structured interview was conducted to explore the factors affecting the self-management support behavior provided by pediatric nurses during transition preparation for AWEs. Semi-structured interviews in mixed-methods research can provide more in-depth and personalized data, aiding in a comprehensive understanding of the complexities surrounding the facilitators and barriers of evidence-based practice. The interview scope was designed according to the theoretical domains framework (TDF) [27]. This is an interview outline coding designed by Michie et al. [28] to find the most comprehensive behavior change theory to guide behavioral intervention research. The TDF includes 12 behavior characteristics domains: knowledge, skills, social/professional role and identity, self-efficacy, outcome expectations, motivation and goals, memory, attention and decision-making process, environmental factors, social influence, emotions, and behavioral norms. The 10.13039/100009593TDF domain characteristics are also in line with Modi et al.'s [9] definition of the category of child self-management support.

In explanatory sequential mixed methods design, one crucial area is that the quantitative results can inform the sampling procedure and point toward the types of qualitative questions to ask participants in the second phase [19]. Therefore, the design of the interview outline content was based on the results of the first phase of quantitative research, and the items with low readiness scores or significant variations were used as the setting focus of the interview topic. The interview outline was determined through discussion with experts in the field of children's epilepsy treatment and care, and the formal interview outline was formed after two rounds of revision. After the preliminary formulation of the interview outline, the researcher conducted a pre-interview with one nurse and adjusted the outline according to the results. The final version of the interview outline is shown in Table 1.

**Table 1:** Semi-structured interview guide.

Theoretical domain framework field	Outline of the interview
Knowledge	Do you know the summary of evidence related to AWEs' self-management support in transitional care for adolescents with chronic diseases? What challenges does this evidence pose to your existing body of knowledge?
Skills	What are the difficulties in implementing the summarized evidence? What specialized training is required?
Social/professional roles and identity	What conflicts does self-management support for transition preparation, according to this summary of the evidence, have with current work processes and norms? Which other staff were involved?
Self efficacy	What do you think of your contribution and ability to implement this evidence in your work?
Expectation of outcome	What are the benefits of implementing this evidence at work, from your perspective and the perspective of AWEs and caregivers? What are the disadvantages?
Motivation and aims	What role do you think nurses play in self-management support in AWEs? What do you think of this role?
Decision making process	How do you decide on self-management support based on evidence and trade-offs when dealing with AWEs' transition preparation services in complex situations (or when faced with different interventions)?
Environmental factors	What help do you need from the department or hospital to implement this evidence in your work?
Social impact	What are the impacts of healthcare institutions and policies, community, school, and family resources on promoting evidence on self-management during a transition in AWEs?
Emotion	How do you help AWEs and caregivers understand and accept evidence-based care recommendations when emotionally disturbed?
Code of conduct	When applying clinical evidence, how do you balance scientific evidence with individual patient differences and exceptional circumstances?
Characteristic of behavior	How do you think nurses can continue to develop and improve their ability to apply self-management supporting evidence in the AWEs transition preparation service?

The semi-structured interview allowed participants to discuss each issue freely based on their experience. Samples with diverse sociodemographic characteristics were chosen to understand better the perspectives of individuals from different demographic backgrounds on evidence-based practice. Interviews were conducted face-to-face though onsite and online by two trained qualitative researchers. Before the interview, the interviewees were contacted to explain the purpose, method and content, to promise strict confidentiality and anonymity, obtain the interviewees' consent, and agree on the interview time. On the day before the interview, online video or face-to-face communication was conducted to explain the evidence summary completed in the early stage and to answer any questions. The interview was conducted in an independent and quiet office on the ward. The interview was recorded with the interviewee's consent, and the critical content and non-language data were recorded. Interviews lasted 10–25min and the recording was transcribed within 48 h. The general information of the interviewees as well as their expressions and movements were analyzed.

### 3.3 Ethical considerations

This research was approved by the Ethics Committee of the local Hospital. To protect confidentiality during and after the research, all personal information was maintained and shared only in de-identified form.

## 4 Data analysis

### 4.1 Quantitative phase

The two authors from phase 1 verified and checked all the data and then imported it into SPSS 18.0 and SAS 9.0 for statistical analysis. Frequency, constituent ratio, and mean ± standard deviation were used for descriptive analysis.

### 4.2 Qualitative phase

After the interviews, the data were analyzed using Colaizzi's 7-step method [29]: 1. transcribe the voice data and carefully combine the keywords of the interview records and the interviewees' expression, tone, eyes, silence time, and body movements; 2. absorb important statements; 3. code recurring meaningful ideas; 4. pool the coded views; 5. write a detailed, non-missing description; 6. identify similar views and synthesize the theme concept; and 7. return to the study subjects for verification. After the interviews, all data were comprehensively analyzed to reflect the barriers and facilitators of evidence application by the interviewees. To verify saturation, two new participants were recruited. The results contained all the important codes and topics put forward by the newly included participants, so it is considered that the samples at this stage are suitable [30].

## 5 Results

### 5.1 Quantitative results

The education background of all respondents was 6 (4.76 %) college degree, 89 (70.63 %) had a bachelor's degree, and 31 (24.60 %) had a master's degree or above. There were 15 nursing managers (11.90 %), 21 specialist nurses (16.67 %), and 90 nurses (71.43 %). In terms of awareness of knowledge and skills of evidence application, no person did not know; 98 people (77.78 %) knew some evidence, and 28 (22.22 %) knew and could apply evidence. In terms of participation in evidence-based practice, 31 (24.60 %) participated and 95 (75.40 %) did not. CREBNA scores: the total score of all items in the scale was 127.06 ± 13.45, and the total scores of the three subscales of evidence, organizational environment, and promoting factors were 49.98 ± 5.17, 36.68 ± 3.76, and 40.39 ± 4.31, respectively (Table 2). Of the 148 questionnaires distributed, a total of 126 questionnaires were collected（The response time of less than 180 s and ≥ five regular answers were excluded.), a valid return rate of 85.14 %.

**Table 2:** CREBNA score (mean ± standard deviation).

Items		Score (±SD)	Rank
1	Evidence subscale (12 Items)	49.98（5.17）	
1.1	The sources of evidence are reliable	4.18（0.58）	8
1.2	The evidence was assessed through a rigorous quality assessment process	4.27（0.62）	6
1.3	Evidence is appropriate for patients/providers in settings where evidence-based practice programs will be implemented	4.21（0.45）	7
1.4	The screening of evidence combines the work experience and professional judgment of clinical nursing staff	4.48（0.29）	2
1.5	The screening of evidence took into account the needs of the patient	4.61（0.26）	1
1.6	The application of evidence can promote the rehabilitation of patients and directly or indirectly improve the outcome of patients	3.76（0.21）	11
1.7	The implementation of evidence can improve the quality of medical/nursing services	4.40（0.05）	4
1.8	The evidence was in accordance with national policies, laws and regulations	4.32（0.51）	5
1.9	The screening of evidence took full account of the current medical conditions and medical level	4.46（0.33）	3
1.10	The content involved in the evidence is within the scope of responsibility of doctors/nurses, and they can intervene in corresponding ways	3.89（1.05）	10
1.11	I am willing to accept this evidence into clinical practice, which is in line with my self-requirements and values	3.31（0.56）	12
1.12	Evidence has been translated into forms that are easy to disseminate, understand, and apply, such as processes, practice manuals, and program publicity posters	4.09（0.44）	9
2	Organizational environment subscale (9 items)	36.68（3.76）	
2.1	Leaders are good at actively exploring and improving clinical work	4.13（0.53）	4
2.2	The leader has a good influence and we are willing to carry out her/his advice or orders	4.25（0.46）	3
2.3	Leaders can allocate human resources according to clinical work	4.00（0.22）	5
2.4	The leader has good communication and coordination skills	4.62（0.27）	1
2.5	Leaders can listen to our views and opinions	4.39（0.32）	2
2.6	I am willing to try new clinical nursing processes, methods, techniques, etc.	3.78（0.66）	8
2.7	I have a good ability to execute the tasks assigned by my superiors	3.72（0.72）	9
2.8	My team members are able to coordinate and work together to achieve specific goals	3.98（0.09）	6
2.9	The ward in which I worked had the cultural atmosphere and workflow of multidisciplinary cooperation	3.81（1.02）	7
3	Facilitating factors subscale (10 items)	40.39 ± 4.31	
3.1	Facilitators with extensive expertise and clinical experience in the evidence-based practice team	3.91（0.86）	6
3.2	The evidence-based practice team has facilitators who can develop practical evidence-based practice programs	4.00（0.67）	5
3.3	I think that the upcoming evidence-based practice has included all relevant people (such as researchers, doctors, nurses, and other multidisciplinary team members).	3.85（1.01）	7
3.4	Incentives to engage in evidence-based practices (e.g., job prospects, learning opportunities, group honors, remuneration, etc.)	3.76（0.44）	10
3.5	I have various forms of training courses related to evidence-based practice programs (e.g., lectures, video lectures, seminars, hands-on exercises)	3.80（0.33）	9
3.6	I have the opportunity to participate in the decision-making of ward-related affairs (making/changing work flow, resource allocation, personnel arrangement, etc.).	4.53（0.36）	1
3.7	The upcoming evidence-based practice project was supported by the senior leadership (hospital/nursing department)	4.22（0.72）	3
3.8	My ward has the information resources needed to carry out evidence-based practice (medical data, software development technology, technical staff support, etc.)	3.82（0.25）	8
3.9	We have a feedback system to optimize the practice plan according to the feedback of clinical nurses and patients	4.19（0.47）	4
3.10	I have a plan to disseminate evidence-based practice (to disseminate current evidence to other hospitals/wards)	4.31（0.65）	2
Total scale		127.06（13.45）	

### 5.2 Qualitative results

A total of 15 nurses participated in the semi-structured interview, including one male and 14 female. The average age was 34.33 ± 4.78 years old and the average number of working years was 9.23 ± 4.55; 11 (73.33 %) had a bachelor's degree, three (20.00 %) had a graduate degree, and one (6.67 %) had a college degree. There were four senior professional titles (26.67 %), nine intermediate titles (60.00 %), and two junior titles (13.33 %). There were four head nurses (26.67 %), two specialist nurses (13.33 %), seven clinical team leaders (46.67 %), and two nurses (13.33 %).

Among the topics discussed during the interviews, the barriers were: insufficient knowledge reserve, lack of auxiliary skills, lack of incentives for evidence-based practice, an imperfect interdisciplinary cooperation platform, heavy workload, inappropriate training methods, and mismatched information tools. Facilitators were: patients and families with obvious needs, an excellent organizational atmosphere, and an apparent willingness to reform.

### 5.3 Emergence of qualitative and quantitative data

The researcher combined the two databases of quantitative and qualitative results. This is the integration point in an explanatory sequential design [19]. Four main themes based on the Knowledge-To-Action framework (KTA) emerged from the integrated findings. These included:

(1) organization level, which in turn included lack of incentive mechanism for evidence-based practice, imperfect interdisciplinary cooperation process, and lack of information auxiliary aids;
(2) operational level, which investigated a lack of exemplary evidence-based practice, high time pressure, and gaps in patient and family understanding of evidence;
(3) personal level, which included a lack of evidence-based and professional knowledge, professional autonomy or a shared decision-making role, and dependence on habitual clinical behavior;
(4) promoting factors including leadership commitment, self-management identity, significant needs for transitional services and a child- and family-centered care culture.

**Fig. 1:**
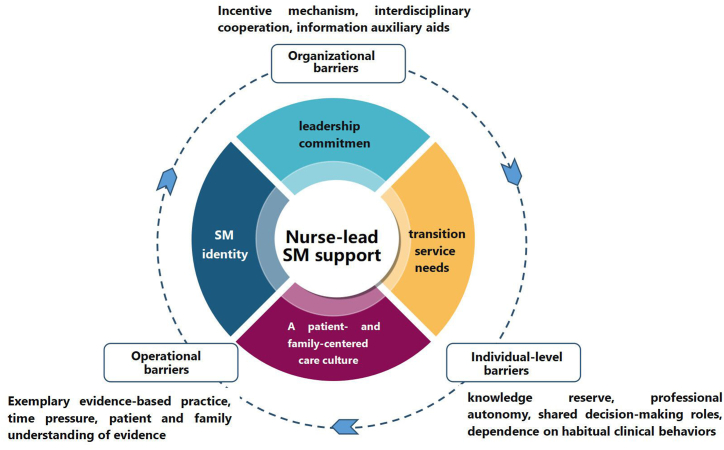
Conceptual model of Barriers and facilitators factors.

The outcomes of the emergence of the qualitative and quantitative data are shown as a Joint display (Supplementary Table 3). We created a loose conceptual model based on KTA, where barriers were classified and visualized at the organizational, operational, and individual levels before evidence application. Facilitating factors were selected as the central action, which links the main results (Fig. 1).

## 6 Discussion

### 6.1 The implementation of evidence-based practice of nurse-led self-management support in transition preparation is feasible

In the field of evidence-based medicine, the application of evidence “builds a bridge between theory and practice” [31]. Assessment of evidence-based nursing practice readiness helps to identify influencing factors in the practice process, select the best intervention before evidence application, and promote evidence transformation [31]. This study used China's first evidence-based nursing practice readiness assessment scale to evaluate current practice. The results indicated that teams have good readiness and capacity for implementation of evidence-based nursing practice Compared with the norm [23,32]. However, the facilitators should be exploited for better implementation.

Leadership commitment should be a focus in evidence-based practice during transition preparation self-management practice. Leadership commitment refers to the attitude and willingness of the leader to be loyal and invested in the organization, which emphasizes the degree to which the team members identify with the leader's behavior and role [33]. In the organizational environment dimension of the CREBNA scale, the average score of items 2.1–2.5 was >4, indicating that good practice by nursing managers can positively impact staff confidence in evidence-based practice. As found during the interviews with pediatric nurses, hospital managers should fully recognize the influence of leadership commitment on evidence-based practice during transition preparation for AWEs, improve the evidence-based literacy of nursing managers, and provide more opportunities for nursing staff to integrate into evidence-based practice, which is conducive to improving the connection between pediatric nurses and hospitals. Similarly, paying attention to the match between the actual conditions of nurses and managers is also an essential factor in improving evidence-based self-management support during transition preparation for AWEs.

A good organizational culture will bring more sense of practical achievement to nurses. Pediatric nurses' recognition of the importance of self-management and the need for transition preparation services is key to developing evidence-based practice. In the evidence dimension of the CREBNA scale, evidence screening combined the work experience of clinical nurses and the needs of patients, and every score was above 4. This indicates both that pediatric nurses have a good recognition of the best evidence and audit indicators and that clinical practitioners have recognized the research topic of self-management support around transition preparation. Therefore, researchers and pediatric nurses should establish close cooperation to promote the sharing and application of evidence of self-management during transition. Investigators can work with clinical practitioners to ensure that research objectives and methods are aligned with real-world clinical settings. At the same time, pediatric nurses can provide practical experience feedback for research and help researchers better understand the challenges of transitional preparation and the individualized needs of patient self-management.

The culture of pediatric care centered on children and families is conducive to applying evidence. The emphasis is on the participation of children and families, such as evidence “establishing a workflow for patients and families to participate in self-management implementation, feedback, and evaluation” [[34], [35], [36]]; “family caregivers knew the acute seizure action plan” [3]; “Model caregivers' daily healthy behaviors” [37]. Of a critical review that evaluated factors associated with self-management, individual and family-focused factors were the most commonly studied predictors [13]. Child-and family-centered pediatric nursing runs through Chinese pediatric nursing textbooks, and it is also the principle of children's chronic disease management in institutions where the evidence of this study is applied. Given the developmental life stage and the unique challenges of living with epilepsy, it is necessary to develop the best evidence-based cooperation strategies between family and AWEs, design quantitative tools for family caregiver participation, and carry out longitudinal research on patient participation level to promote the best tacit understanding of self-management behavior between AWEs and family caregivers.

### 6.2 The incentive mechanism, interdisciplinary cooperation, and information auxiliary aids at the level of the evidence implementation system need to be optimized

#### 6.2.1 Enrich the incentive for evidence-based activities in pediatric nursing and establish an applicable evidence-based practice evaluation method

This study constructed a framework of semi-structured interviews based on TDF, designed the interview content combined with the quantitative research results, and explored the barriers at the system level. In the quantitative research results, the score of incentive policies to promote participation in the facilitators dimension was 3.76 ±0 .48, 10/10. Therefore, the semi-structured interview was set up in the field of social influence and behavioral characteristics to explore the factors encouraging pediatric nurses to carry out evidence-based practice. At the same time, specialist nurses were considered to have the advantages of participating in decision-making, identifying motivation, and promoting team development in clinical work [38], so they were included in the interview population. The results found that nurses' identification with the meaning of AWEs self-management support was essential. Pediatric nurses can standardize clinical practice following the best evidence. However, if the self-management intervention of transition preparation benefits cannot be immediately highlighted, especially under the condition of increasing workload and when there is no incentive mechanism such as rewards, promotion, or professional development opportunities, the enthusiasm of pediatric nurses to participate in evidence-based practice may be reduced. Management should increase the diversity of incentives for self-management during transition, such as material rewards, training opportunities, and career development. According to the results, an appropriate performance evaluation and incentive mechanism for the transition process were established, attention was paid to the benefits of nursing reform, and more pediatric nurses were encouraged to participate in evidence-based nursing practice of self-management during the transition.

#### 6.2.2 Interdisciplinary cooperation to improve the efficiency of self-management support

Both NICE guidelines [39] and expert consensus [40] suggest that self-management in transition preparation should be carried out by interdisciplinary teams, including child and adult health care workers, psychotherapists, social workers, and school and community workers. In this study, the scores of 2.9 and 3.3 were 3.81 ± 0.50, 7/9 and 3.85 ± 0.42, 7/10. Pediatric nurses proposed that the focus of self-management support of AWEs and families differed due to the wide range of specialties involved as well as patient needs. This means that intervention can be missed or delayed in interdisciplinary fields during transition preparation. For example, for sleep assessment for AWEs, nurses play the most direct role in collecting information, but professional intervention must be completed by clinicians. If psychological factors cause the sleep disorder for AWEs, patients must be referred to a psychological clinic. However, sleep problems are often left untreated due to a lack of professional assessment and referral. In addition, knowledge of epilepsy should be popularized among primary and secondary school teachers, and training on basic seizure treatment techniques should be carried out [41]. Nursing administrators expressed needing clarification about the continuous cooperation between hospitals and schools. To overcome these challenges, interdisciplinary collaboration in self-management support during transition preparation for AWEs must establish clear communication channels and roles. This fosters an open and inclusive culture of collaboration, providing interdisciplinary education and enhancing the collaborative skills of team members.

#### 6.2.3 Information auxiliary aids help pediatric nurses to implement individualized support strategies

NICE guidelines [39] and a systematic review [42] suggest that self-management, resource management and decision support tools should be available. Juvenile epilepsy is a chronic disease with significant individual variability, so each patient may have different treatment and care needs. Self-management software can help AWEs and families improve their skills by providing rich information resources. At the same time, information tools can collect patients' health data, such as seizure frequency, medication use and triggers, and provide real-time monitoring and feedback to achieve more precise support. It is vital to ensure that the design and functionality of the system meet the needs of AWEs and families, provide an easy-to-understand and friendly interface, and meet the expectations of different disciplines for disseminating information in transition preparation, ensuring that the platform is reliable and secure.

### 6.3 Cost-effectiveness, exemplary case of evidence-based practice, and patient-family participation contribute to the continuous application of evidence in clinical practice

In this study, the scores of items 3.1, 2.6, and 1.6 in the quantitative research were 3.91 ± 0.38, 6/10, 3.78 ± 0.41, 8/9, and 3.76 ± 0.46, 11/12, respectively. During the interview, the nurses mentioned that “although there is great support for AWEs self-management support as recommended by evidence, there are some concerns about the work time and cost-effectiveness in clinical practice.” In the neurology department, AWEs' self-management support work is only part of the clinical work, which includes drug administration, monitoring, safety assurance, medical history recording, and primary nursing. Implementation strictly under evidence recommendations and standards will take a certain proportion of time; clinical nurses focus on whether nurses can obtain the expected results. Therefore, it is recommended to carry out integrated knowledge translation research at the organizational level and incorporate the perspectives of clinical nurses, AWEs, and other stakeholders, and improve evaluation of the cost-effectiveness of evidence implementation. This should improve the confidence of pediatric nurses in carrying out transition preparation services.

At the same time, the peer effect of nurses can promote knowledge sharing and provide support [38]. Practice managers can encourage more pediatric nurses to participate in evidence-based practice through the role model of evidence implementor and practice demonstration.

Information should be presented concisely and easily understandable for AWEs and families, such as charts, images, and graphical tools [39]. Developing personalized patient education plans and establishing active doctor-patient communication and participation [3] helps implement evidence-based practice during transition preparation.

### 6.4 The improvement of professional ability and optimization of professional characteristics at the individual level can promote evidence-based implementation

In the quantitative research phase, items 3.5 (3.80 ± 0.41, 9/10) and 1.10 (3.89 ± 0.39, 10/10) showed that the training resources of evidence-based practice and the scope of responsibility of doctors and nurses had low scores. Through interviews, the content of the quantitative results was explained and expanded, and the judgment of pediatric nurses in evidence-based and transitional services knowledge reserve, participation in shared decision-making roles and lack of professional characteristics was obtained. The organization of learning and training is an essential factor influencing the ability to use evidence-based nursing practice [27]. Although the hospital has standardized, evidence-based nursing and transition preparation and carried out unified training and sharing exchanges, the application and self-management skills in evidence-based nursing practice combined with pediatric nurses still need to be improved, and more targeted guidance is needed. Most pediatric nurses in this study recognized the importance of establishing AWE- and family-centered shared decision-making (SDM). However, they lacked awareness of implementing assisted decision-making in self-management support work. Nurses have both positive and negative opinions about nurse-patient SDM practice because the inherent thinking and behavior model of doctor-led decision-making in clinical practice hinders nurses from adapting to the requirements of SDM. Combined with the core role of nurses in transition preparation, pediatric nurses need to further develop their skills in participating in SDM to better play the role of decision mentor and coordinator in self-management support.

Regarding occupational characteristics, the significant barriers were a lack of occupational autonomy and dependence on habitual clinical behaviors. Nurses' professional autonomy enables them to make independent decisions in clinical practice, and formulate the best care plan according to the latest evidence, combined with the individual situation and preferences of patients [38]. When nurses are empowered with greater autonomy and decision-making, they are more motivated to actively seek out the latest research evidence, improve practice, and promote evidence-based practice [23,38]. The practice of transition preparation requires interdisciplinary collaboration, and nurses must share responsibility with other professionals. Item 1.11, about nurses' willingness to accept the application of such evidence to clinical practice, scored the lowest in the same dimension (3.31 ± 0.56, 12/12). The description of clinical behavior characteristics by pediatric nurses in the qualitative data expanded the results of nurses' reliance on habitual clinical behavior. Implementing the reform will challenge nurses' ingrained habits during transition practice. When problems occur, the tendency to use original work habits will increase [23]. Changing nurses' habitual clinical behavior takes time and effort. Incentive systems, clinical decision-support tools, self-reflection, continuous supervision, and feedback on self-management during transition may change this. At the same time, barriers sometimes become facilitators. Once new processes are applied in clinical practice and new habits are formed, the implementation rate of evidence-based practice programs will increase.

## 7 Strengths and limitations

This is the first mixed methods study to explore the barriers and facilitators of self-management support of adolescents with epilepsy based on evidence-based practice among nurses. The strengths of this study include the methodological and multicenter approach. Trustworthiness was achieved through the triangulation of data and peer debriefing of the qualitative data. However, study limitations are evident. First, the sites were all located in southwest China, and regional differences may make the study results one-sided. Second, due to the limitation of the sample size, this study could not find relevant factors affecting the score of evidence-based clinical readiness. In the future, the number of application sites and sample size can be increased to describe the current situation more fully. Finally, we could not rule out selection bias due to the sampling strategy used in the semi-structured interviews nor information bias due to self-reporting. Future research could consider a longitudinal approach based on the transition preparation stage to further explore the long-term behavioral characteristics of pediatric nurses in self-management support for adolescents with chronic diseases.

## 8 Conclusion

Encouraging self-management support among nurses supporting adolescents with chronic diseases in the transitional stage is a complex issue. Multiple factors affect the program's durability. In practice, it is necessary to create a cultural atmosphere of multi-stakeholder participation according to the personalized needs of self-management in transition. It is important to give full play to the advantage of leadership commitment in the organization, optimize interdisciplinary cooperation and evidence-based incentive mechanisms, carry out excellent evidence-based case demonstration and multidisciplinary training, establish a standard process for pediatric nurses to participate in patients' self-management decision-making aids, and improve project quality control to promote evidence-based action to maximize the effectiveness of clinical practice.

## What is already known

●It is vital to develop self-management skill in transition preparation for adolescents with neurological disease, yet current support approaches are inadequate.
●Development of high-quality pediatric epilepsy self-management interventions depends on best evidence-based practice.
●Nurses play a key role in transition preparation services and self-management support.

## What this paper adds

●The evidence-based practice of nurse-led self-management in adolescents with epilepsy during the transition period included ten barriers and four facilitators though a mixed-methods study.
●A conceptual model based on KTA is beneficial for developing self-management support strategies for adolescents with epilepsy and families.

## Funding

This research was funded by the Chongqing Science and Health Joint Medical Research Project (2022GDRC007); Program for Youth Innovation in Future Medicine, 10.13039/501100004374Chongqing Medical University (W0008).

## Ethics approval and consent to participate

This research was approved by the Ethics Committee of the hospital conducting the research (No. 2022–32). To protect confidentiality during and after the research, all personal information was maintained and shared only in de-identified form.

## Data availability statement

The datasets generated and analyzed during the present study are available from the corresponding author on reasonable request.

## CRediT authorship contribution statement

**Cui Cui:** Writing – original draft, Formal analysis, Conceptualization. **Shuangzi Li:** Formal analysis, Data curation. **Hengyu Zhou:** Investigation. **Wenjin Chen:** Data curation. **Changmin Xiao:** Data curation. **Mingping Fan:** Data curation. **Xianlan Zheng:** Conceptualization.

## Declaration of competing interest

The authors declare that they have no known competing financial interests or personal relationships that could have appeared to influence the work reported in this paper.
