# Supplementary material for: Barriers and facilitators of nurse-led self-management support for adolescents with epilepsy: A mixed-methods study in transition preparation
Source: Heliyon. 2024 Jun 26;10(13):e33774. doi: 10.1016/j.heliyon.2024.e33774 (PMC11283166; doi:10.1016/j.heliyon.2024.e33774)
Supplement: Multimedia component 1 [file mmc1.docx]

**Supplementary Figure 1.** Algorithm for the mixed methods expanded coverage design used in the study


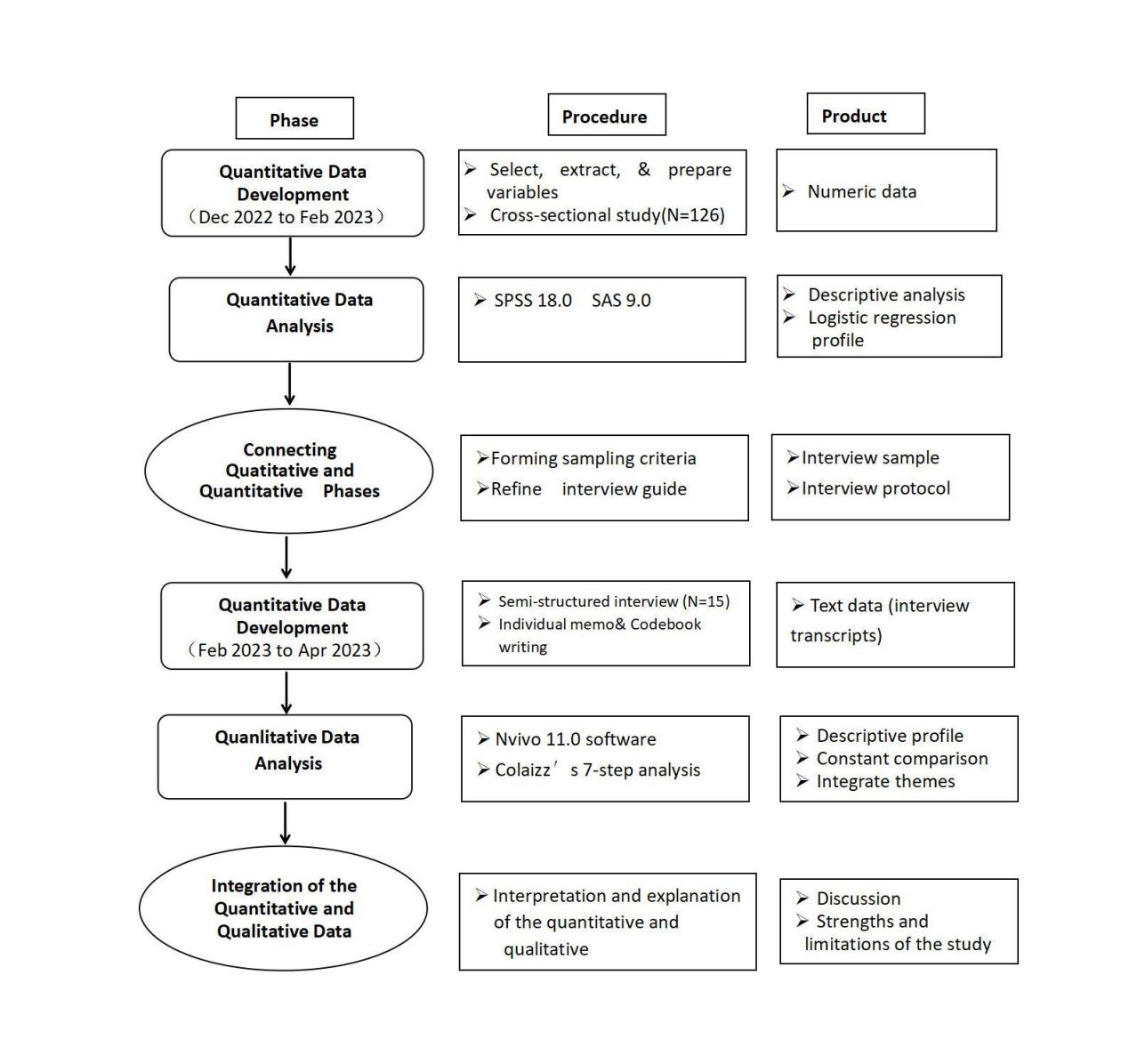


**Supplementary Table 1**  **General information questionnaire**

| **Item** | **Variable** |
| --- | --- |
| **Gender** | **Male** |
|  | **Female** |
| **Years of work(year)** | **< 3** |
|  | **3-7** |
|  | **> 7** |
| **Educational Background** | **Associate Degree** |
|  | **Bachelor's Degree** |
|  | **Graduate Degree** |
| **Position** | **Nursing managers** |
|  | **Specialist nurses** |
|  | **Nurses** |
| **Awareness of Applying Knowledge and Skills to Evidence** | **Unaware** |
|  | **Being aware of some** |
|  | **Being aware and capable of Applying** |
| **The necessity of conducting evidence-based practice** | **Yes** |
|  | **No** |
| **Have you been involved in evidence-based practice?** | **Yes** |
|  | **No** |
| **Willing to join the evidence-based nursing practice team** | **Yes** |
|  | **No** |

**Supplementary Table 2 Clinical readiness of evidence-based nursing assessment (CREBNA) questionnaire**

| **Items** | | entirely consistent | Basically  consistent | Partly  consistent | Basically  inconsistent | completely inconsistent |
| --- | --- | --- | --- | --- | --- | --- |
| **1** | **Evidence subscale (12 Items)** |  |  |  |  |  |
| **1.1** | **The sources of evidence are reliable** |  |  |  |  |  |
| **1.2** | **The evidence was assessed through a rigorous quality assessment process** |  |  |  |  |  |
| **1.3** | **Evidence is appropriate for patients/providers in settings where evidence-based practice programs will be implemented** |  |  |  |  |  |
| **1.4** | **The screening of evidence combines the work experience and professional judgment of clinical nursing staff** |  |  |  |  |  |
| **1.5** | **The screening of evidence took into account the needs of the patient** |  |  |  |  |  |
| **1.6** | **The application of evidence can promote the rehabilitation of patients and directly or indirectly improve the outcome of patients** |  |  |  |  |  |
| **1.7** | **The implementation of evidence can improve the quality of medical/nursing services** |  |  |  |  |  |
| **1.8** | **The evidence was in accordance with national policies, laws and regulations** |  |  |  |  |  |
| **1.9** | **The screening of evidence took full account of the current medical conditions and medical level** |  |  |  |  |  |
| **1.10** | **The content involved in the evidence is within the scope of responsibility of doctors/nurses, and they can intervene in corresponding ways** |  |  |  |  |  |
| **1.11** | **I am willing to accept this evidence into clinical practice, which is in line with my self-requirements and values** |  |  |  |  |  |
| **1.12** | **Evidence has been translated into forms that are easy to disseminate, understand, and apply, such as processes, practice manuals, and program publicity posters** |  |  |  |  |  |
| **2** | **Organizational environment subscale (9 items)** |  |  |  |  |  |
| **2.1** | **Leaders are good at actively exploring and improving clinical work** |  |  |  |  |  |
| **2.2** | **The leader has a good influence and we are willing to carry out her/his advice or orders** |  |  |  |  |  |
| **2.3** | **Leaders can allocate human resources according to clinical work** |  |  |  |  |  |
| **2.4** | **The leader has good communication and coordination skills** |  |  |  |  |  |
| **2.5** | **Leaders can listen to our views and opinions** |  |  |  |  |  |
| **2.6** | **I am willing to try new clinical nursing processes, methods, techniques, etc.** |  |  |  |  |  |
| **2.7** | **I have a good ability to execute the tasks assigned by my superiors** |  |  |  |  |  |
| **2.8** | **My team members are able to coordinate and work together to achieve specific goals** |  |  |  |  |  |
| **2.9** | **The ward in which I worked had the cultural atmosphere and workflow of multidisciplinary cooperation** |  |  |  |  |  |
| **3** | **Facilitating factors subscale (10 items)** |  |  |  |  |  |
| **3.1** | **Facilitators with extensive expertise and clinical experience in the evidence-based practice team** |  |  |  |  |  |
| **3.2** | **The evidence-based practice team has facilitators who can develop practical evidence-based practice programs** |  |  |  |  |  |
| **3.3** | **I think that the upcoming evidence-based practice has included all relevant people (such as researchers, doctors, nurses, and other multidisciplinary team members).** |  |  |  |  |  |
| **3.4** | **Incentives to engage in evidence-based practices (e.g., job prospects, learning opportunities, group honors, remuneration, etc.)** |  |  |  |  |  |
| **3.5** | **I have various forms of training courses related to evidence-based practice programs (e.g., lectures, video lectures, seminars, hands-on exercises)** |  |  |  |  |  |
| **3.6** | **I have the opportunity to participate in the decision-making of ward-related affairs (making/changing work flow, resource allocation, personnel arrangement, etc.).** |  |  |  |  |  |
| **3.7** | **The upcoming evidence-based practice project was supported by the senior leadership (hospital/nursing department)** |  |  |  |  |  |
| **3.8** | **My ward has the information resources needed to carry out evidence-based practice (medical data, software development technology, technical staff support, etc.)** |  |  |  |  |  |
| **3.9** | **We have a feedback system to optimize the practice plan according to the feedback of clinical nurses and patients** |  |  |  |  |  |
| **3.10** | **I have a plan to disseminate evidence-based practice (to disseminate current evidence to other hospitals/wards)** |  |  |  |  |  |

**Supplementary Table 3. Comparison and merging of Barriers and facilitators of self-management support based on evidence-based practice (Joint display of data)**

| **Domain** | **Subtheme** | **Quantitative** | **Qualitative quotes** | **Inferences** |
| --- | --- | --- | --- | --- |
| **Organization level** | **Lack of incentive mechanism for evidence-based practice** | **Item3.4**  **（3.76±0.48; 10/10）** | ***“We spend so much time and effort keeping up with the latest research and best practices, but it feels like there's no recognition or reward for our efforts.” (N13)***  ***“It's disheartening to see colleagues who don't prioritize evidence-based care being treated the same as those of us who strive to provide the best possible care based on current research.” (N8)*** | **Explanation**  **The lack of time, spirit, or material rewards for evidence-based practice in the qualitative data explains the lower scores of incentive policies in the quantitative results.** |
|  | **Imperfect interdisciplinary cooperation process** | **Item2.9**  **（3.81±0.50; 7/9)**  **Item3.3**  **（3.85±0.42；7/10）** | ***“It feels like there is a disconnect between healthcare professionals from different disciplines, making it difficult to ensure a smooth and coordinated transition for patients, which hinders the care support.” (N2)***  ***“The incomplete interdisciplinary collaboration mechanism also adds unnecessary stress and workload on nurses.” (N5)***  ***"Sometimes in evidence execution, such as sleep assessment, AWEs have a lot of problems, but do not know who to contact to solve them" (N14)*** | **Explanation**  **The distress of not running smoothly across disciplines in the qualitative data explains the lower scores for multidisciplinary collaboration and personnel composite in the quantitative results.** |
|  | **Lack of additional information tools** | **Item3.8**  **（3.81±0.40；8/10）** | ***“The lack of effective and user-friendly digital tools makes it challenging for patients to track and manage their health conditions independently.” (N12)***  ***“Better information systems are needed to meet the evidence requirements for personalized self-management support and to ensure the quality of services.” (N1, N4)*** | **Explanation**  **The need for an information platform in the qualitative data explains the lower score of information resources required for evidence-based practice in the quantitative results.** |
| **Operational level** | **Lack of demonstration of good evidence-based practice** | **Item3.1**  **（3.91±0.38；6/10）** | ***“The scarcity of exemplary evidence-based practice models in self-management hampers our ability to provide tailored and effective care that aligns with patients' diverse needs and preferences.” (N7)*** | **Expansion**  **The learning needs of typical evidence-based practice cases in the qualitative data extended the lower scores given to evidence-based practice facilitators in the quantitative results.** |
|  | **High time pressure** | **Item2.6**  **（3.78±0.41；8/9）** | ***“I understand how important self-management is for patients, but I don't have a lot of time to think and switch what works best. It's very frustrating!” (N9)***  ***“I'm swamped with work and barely had time to understand and apply best practices and just wanted to get my work done before the end of the day.” (N3)*** | **Expansion**  **The distress of pediatric nurses with the existing workload in the qualitative data extends the low scores of practitioners willing to try new processes in the quantitative results.** |
|  | **Gaps in patient and family understanding of evidence** | **Item1.6**  **（3.76±0.46；11/12）** | ***“I try to recommend families to use medication adherence tools (e.g., pill boxes with alarm clocks, phone apps, bundles of memories with specific daily activities, etc.), but they don't see the need; they are confident that taking their medication is more important than eating and therefore don't need any complementary or reinforcing approaches” (N12)*** | **Expansion**  **The distress of pediatric nurses with the existing workload in the qualitative data extends the low scores of practitioners willing to try new processes in the quantitative results.** |
| **Personal level** | **Lack of evidence-based and professional knowledge** | **Item3.5**  **（3.80±0.41；9/10）** | ***“Due to the lack of education and knowledge in evidence-based practice and transitional care, I always had gaps in understanding and implementing evidence, such as the content of evidence-based health education, which I could not give the most appropriate explanation to the patient and family” (N11)*** | **Explanation**  **In qualitative data, the reflection of pediatric nurses on the lack of evidence-based knowledge and transition service-related knowledge was given, and the low scores of related training courses in quantitative data were explained.** |
|  | **Lack of professional autonomy** | **Item2.7**  **（3.72±0.39；9/9）** | ***"In nurse-led self-management support, I think it is not good to practice evidence simply by executing doctor's advice and executing the requirements of superiors, because in the management of patients' health literacy, nurses need to constantly interact with patients and families, but the authority of nurses' practice limits the results of interaction and reduces the scope of evidence application" (N9)***  ***“When AWEs are found to need psychological education, nurses are required to promptly refer patients to the department of psychology, but the current situation is that a referral order is required from a doctor first (N14).”*** | **Expansion**  **The contradiction between pediatric nurses expressing their work authority and self-management support in the qualitative data extends the results of lower executive scores in the quantitative data.** |
|  | **Lack of shared decision-making role** | **Item1.10**  **（3.89±0.39；10/10）** | ***There are times when I struggle to advocate for AWEs and families' preferences and concerns based evidence during discussions with doctors, leading to compromised shared decision-making." (N6)***  ***“I feel like there's a lack of mutual understanding and respect between doctors and nurses when it comes to shared decision-making about AED guidance, making it challenging to work together for transition services." (N15)*** | **Expansion**  **The experience of pediatric nurses participating in shared decision-making as a family spokesperson in the qualitative data extends the results of the quantitative data in which the evidence can be implemented within the scope of health care responsibilities.** |
|  | **Dependence on habitual clinical behavior** | **Item1.11**  **（3.31±0.56；12/12）** | ***“I would be delight to accept this evidence for clinical application, but I also came to realize that my reliance on habitual clinical behavior may not always be the most effective, but certainly the quickest.” (N5)***  ***The evidence required a concise oral explanation of the SUDEP concept and risks according to the specific needs of AWEs/ caregivers, but I always used to hand them a leaflet, too much explanation, I was worried about causing their psychological burden. (N1)*** | **Expansion**  **The experience of pediatric nurses in the qualitative data with respect to their preferences for clinical habits of behavior extends the results in the quantitative data with lower scores for the application of practitioners willing to accept evidence.** |
| **Promoting factors** | **Leadership commitment** | **Item2.1-2.5**  **（均分4.29±0.38；1/9-5/9）** | ***"This project has been included in the scientific research project of the municipal Health commission, and the head nurse and the nursing department actively coordinate human and material resources and give financial support in the implementation" (N7”***  ***"The head nurse has a postgraduate education background and can give us timely guidance and help in the leadership and implementation of this project" (N12)*** | **Explanation**  **The positive evaluation of pediatric nurses on team organizers in the qualitative data explained the higher scores on the organizational and coordination ability of leaders in the quantitative data.** |
|  | **Self-management identity** | **Item1.4**  **（4.48±0.34；2/12）** | ***"Improving the self-management capacity of AWEs and families is an important issue that should help promote patient health, reduce readmission rates, and reduce the use of health care resources." (N4)***  ***"I would love to provide self-management support and guidance to the AWEs and their families, and would be happy to know that they can make a smooth transition to adult medical institutions with my help" (N9)*** | **Explanation**  **The positive attitude of pediatric nurses towards self-management support in qualitative data was explained, and the high score of evidence screening combined with professional judgment of clinical staff in quantitative data was explained.** |
|  | **Significant needs for transitional services** | **Item1.5**  **（4.61±0.40；1/12）** | ***"In clinical practice, adolescents and families with epilepsy face increasingly specific and varied questions about the transition from pediatric to adult care, and some are willing to pay for this transition" (N11).***  ***"I often have family caregivers describe to me that they have become accustomed to the interaction and medical treatment patterns with pediatric medical staff, and feel anxious and helpless at the thought of transferring to an unfamiliar adult hospital. I am eager to help these families" (N6)*** | **Explanation**  **The experience of nurses on the increased needs of patients and families for transition preparation in the qualitative data, and the higher scores of evidence screening combined with patient needs in the quantitative data were explained.** |
|  | **A child- and family-centered care culture** | **Item1.9**  **（4.46±0.43；3/12）** | ***"There is no barrier to carrying out family-based seizure drills here, we have a more mature way of organizing them" (N2) "We have always advocated the involvement of families and adolescents with chronic diseases in disease management. Many of our brochures are designed for parents, adolescents, and families, which is conducive to the practice of family participatory self-management evidence" (N10).*** | **Explanation**  **Nurses' observation of the way patients and families participate together in the qualitative data, and the evidence screening combined with environmental conditions in the quantitative data were explained.** |
